# Asymmetric Lipid Transfer between Zwitterionic Vesicles by Nanoviscosity Measurements

**DOI:** 10.3390/nano11051087

**Published:** 2021-04-22

**Authors:** Laure Bar, George Cordoyiannis, Shova Neupane, Jonathan Goole, Patrick Grosfils, Patricia Losada-Pérez

**Affiliations:** 1 Experimental Soft Matter and Thermal Physics Group (EST), Department of Physics, Université Libre de Bruxelles, 1050 Brussels, Belgium; laure.bar@ulb.be; 2 Condensed Matter Physics Department, Jožef Stefan Institute, 1000 Ljubljana, Slovenia; georgios.kordogiannis@ijs.si; 3 Physical Chemistry of Surfaces Group, Institut de Recherche de Chimie Paris (IRCP), 75005 Paris, France; shova.neupane@chimieparistech.psl.eu; 4 Laboratory of Pharmaceutics and Biopharmaceutics, Campus de la Plaine, Université Libre de Bruxelles, 1050 Brussels, Belgium; jonathan.goole@ulb.be; 5 Center for Nonlinear Phenomena and Complex Systems, Department of Physics, Université Libre de Bruxelles, 1050 Brussels, Belgium; patrick.grosfils@ulb.ac.be

**Keywords:** solid-supported lipid membranes, lipid transfer, quartz crystal microbalance with dissipation monitoring, phase transitions, atomic force microscopy

## Abstract

The interest in nano-sized lipid vesicles in nano-biotechnology relies on their use as mimics for endosomes, exosomes, and nanocarriers for drug delivery. The interactions between nanoscale size lipid vesicles and cell membranes involve spontaneous interbilayer lipid transfer by several mechanisms, such as monomer transfer or hemifusion. Experimental approaches toward monitoring lipid transfer between nanoscale-sized vesicles typically consist of transfer assays by fluorescence microscopy requiring the use of labels or calorimetric measurements, which in turn require a large amount of sample. Here, the capability of a label-free surface-sensitive method, quartz crystal microbalance with dissipation monitoring (QCM-D), was used to monitor lipid transfer kinetics at minimal concentrations and to elucidate how lipid physicochemical properties influence the nature of the transfer mechanism and dictate its dynamics. By studying time-dependent phase transitions obtained from nanoviscosity measurements, the transfer rates (unidirectional or bidirectional) between two vesicle populations consisting of lipids with the same head group and differing alkyl chain length can be estimated. Lipid transfer is asymmetric and unidirectional from shorter-chain lipid donor vesicles to longer-chain lipid acceptor vesicles. The transfer is dramatically reduced when the vesicle populations are incubated at temperatures below the melting of one of the vesicle populations.

## 1. Introduction

The interfacial properties of lipid-based assemblies are of major importance for the interbilayer lipid transfer/exchange essential in maintaining the function of cell membranes and in many other biologically relevant processes involving the fusion of membranes in cases of viral infection, hormone, and neurotransmitter release [1,2,3]. A substantial amount of the current understanding of the molecular mechanisms involving lipid transfer and exchange between membranes has arisen from studies employing model membrane systems due to the complex nature of membranes in vivo. Interbilayer lipid transfer occurs either through vesicular transport in which large amounts of lipids are transferred via protein-mediated fusion of vesicles or through non-vesicular transport by lipid transfer proteins that shuttle lipid monomers between membranes [4,5].

Unlike protein-mediated processes, spontaneous protein-free lipid transfer remains less explored, partly because it is slower and biologically less relevant and requires larger activation energies to proceed [6,7]. Nevertheless, spontaneous lipid transfer has been observed in biological processes involving lipid metabolism or parasitic infections [8,9], and its understanding is relevant for the design of lipid-based nanocarriers and for elucidating the active role of lipids in biological processes [10,11]. The kinetics of lipid transfer between two bilayer membranes are intimately related to the thermodynamics of lipid mixing and the lateral organization of their components. These eventually depend on the physicochemical characteristics of the constituent membrane lipids, in particular composition, charge, phase, and the membrane shape geometry (bilayer, vesicle). When two bilayer membranes approach each other, spontaneous lipid transfer can take place by means of several mechanisms: (i) transfer of individual lipids from one membrane into another by diffusion through the aqueous medium, (ii) direct lipid transfer between the apposing membranes without exposure to the aqueous medium, which can happen in cases of short intermembrane separations, and (iii) reorganization of the membrane structure into a hemi-fused state, where two proximal leaflets partially fuse using a stalk formation, resulting in a rapid lipid exchange between the opposing membranes through lateral diffusion [11,12,13].

From a purely physicochemical perspective, lipid membranes bearing opposite charges where transfer/exchange proceeds driven by electrostatic interactions, have attracted more attention. The customary methodology for probing spontaneous lipid transfer and exchange is assays involving fluorescently labeled large vesicles at self-quenching concentrations in bulk [14,15]. Apart from bulk methods, surface-sensitive label-free techniques may also be used, such as quartz crystal microbalance with dissipation monitoring (QCM-D) and surface plasmon resonance [16,17]. The former is an acoustic-based technique measuring changes in frequency and energy dissipation of a shear-mode oscillating quartz sensor upon mass adsorption [18]. The shear waves propagate as evanescent waves decaying across the boundary between the sensor and the fluid environment with a penetration depth $\delta =\sqrt{\eta_{L}/\pi f_{n}\rho_{L}}$, depending on the overtone frequency *f_n_*, on the viscosity *η_L_* and density *ρ_L_* of the fluid in contact with the sensor surface. The penetration depth of a 5 MHz shear wave in water is *δ* ∼ 250 nm, rendering QCM-D surface-specific and enabling monitoring changes in the shear viscosity of nanoscale-sized layers. QCM-D has been particularly useful in real-time monitoring of the ‘attachment-transfer-detachment’ processes for oppositely small charged vesicles onto solid-supported lipid bilayers. In particular, it contributed to elucidating the role of charge in the kinetics of the process [16,19,20]. Mechanistically, transfer occurs in both directions between both charged bilayers via monomer transfer or intermediate hemifusion structures [21]. When the lipid molecules have an effective charge, a minimum amount of charged vesicles must adsorb on the supported lipid bilayers (SLB), leading to charge reversal in the latter, indicating that detachment occurs after charge neutralization of the small unilamellar vesicles due to lipid exchange between the two interacting membranes.

Apart from real-time adsorption, the unique sensitivity of QCM-D to mass and energy dissipation changes at the solid-lipid layer-liquid interface enables the detection of phase transformations of solid-supported membrane geometries [22,23,24]. Upon heating, lipid bilayers change from a more viscous and stiffer gel phase to a less viscous and softer liquid-disordered phase. These changes manifest as anomalies in both frequency and dissipation shift signals and, in particular, in their first-order temperature derivatives [25,26]. This feature is advantageous compared to traditional calorimetric methods since it does not require either long temperature equilibration times or a large amount of sample.

Here we assess the capability of QCM-D to detect lipid transfer between nanoscale-sized zwitterionic lipid vesicles at a very small concentration, whose interest lies in their use as mimics for endosomes, exosomes, and nanocarriers for drug delivery [27]. Specifically, we focused on detecting lipid transfer taking place between two vesicle populations of zwitterionic saturated phospholipids differing in their alkyl chain lengths—namely, 1,2-dimyristoyl-sn-glycero3-phosphocholine (DMPC, chain length *n* = 14, melting temperature *T_m_* ∼ 24.5 °C) and 1,2-dipalmitoyl-sn-glycero-3-phosphocholine (DPPC, *n* = 16, *T_m_* ∼ 41.5 °C). The net transfer kinetics between the vesicles were inferred from their changes in phase transition (melting) temperatures with time after the two populations were incubated at a given temperature.

## 2. Materials and Methods

### 2.1. Materials Used

DPPC and DMPC lipids (in powder form) were purchased from Avanti Polar Lipids (Alabaster, AL, USA) and spectroscopic grade chloroform from VWR Chemicals, Leuven, Belgium. The HEPES running buffer was prepared with HEPES powder ≥ 99.5% (Sigma-Aldrich, Overijse, Belgium), NaCl powder ≥ 99% (Sigma-Aldrich, Overijse, Belgium), and NaOH powder (VWR chemicals, Leuven Belgium). The masses of lipids were determined gravimetrically using an analytical balance (AG245, Metter-Toledo, Switzerland) with a precision of ± 0.1 mg. The HEPES buffer solution (pH 7.4) used for the hydration of the dried lipid films was prepared by dissolving 10 mM HEPES and 150 mM NaCl in Milli-Q water (18.2 MΩ). The pH was then adjusted to 7.4 with 1M NaOH solution previously prepared with NaOH powder and Milli-Q water. To this end, drops of NaOH solution were added to the buffer during gentle stirring. The pH value evolution was tracked with a FiveEasy Mettler Toledo pHmeter from Mettler Toledo, Zaventem, Belgium. The buffer was then filtered with 0.2 μm-pore size PTFE membranes and stored at 4 °C until being used.

### 2.2. Lipid Vesicle Preparation

Lipids in powder were dissolved in spectroscopic grade chloroform in a round-bottomed flask. The solvent was subsequently evaporated under a continuous mild flow of N_2_. To avoid any residues of solvent, the lipid films were kept under low pressure overnight. The films were then hydrated with fresh filtered HEPES buffer to 2 mg/ml under continuous stirring for 45 min in a temperature-controlled water bath at 60 °C (well above the melting temperatures of DPPC *T_m_* ~ 41.5 °C and DMPC *T_m_* ~ 24.5 °C). Large unilamellar vesicles (LUVs) were formed by extrusion through filters with different pores of 100 nm for 25 passes.

The extruded solutions were then diluted in HEPES buffer for obtaining desired concentrations. DMPC and DPPC stock solutions were diluted at 0.1 mg/ml (to reach a final concentration of 0.05 mg/ml for each lipid after a 50:50 volume mixing, corresponding to a 0.52/0.48 DMPC/DPPC molar ratio). DMPC and DPPC stock solutions used to inject pure vesicles were directly diluted at 0.05 mg/mL. All solutions were immediately stored in a temperature-controlled incubator (Incu-Line 68R, VWR, Poland; temperature fluctuation ± 0.1 °C) at the desired temperatures (*T* = 16 °C, *T* = 32 °C, or *T* = 50 °C).

### 2.3. Dynamic Light Scattering and Zeta Potential Measurements

The vesicle diameters, polydispersities, and ζ potentials were determined by means of dynamic light scattering (DLS) (Malvern Zetasizer Nano ZS, Malvern, UK). The obtained mean diameters and polydispersity indexes of the samples used are displayed in Table 1.

The mean diameters of DPPC and DMPC vesicles range between 110 and 130 nm, and the maximum recorded polydispersity value is 0.12, suggesting that vesicle dispersions are homogeneous in size. As previously reported by Enoki et al. [28] for DMPC, the vesicles display a slightly larger mean diameter when lipids are in the liquid disordered phase. For each sample, the zeta-potential was determined in the range between −2.8 and 1.1 mV, confirming that DPPC and DMPC vesicles are zwitterionic in the buffer used.

### 2.4. Quartz Crystal Microbalance with Dissipation Monitoring

QCM-D measurements were carried out in a Qsense E4 instrument (Biolin Scientific, Gothenburg, Sweden), which enables monitoring of frequency and dissipation changes, Δ*f*/n and Δ*D*, with n the overtone number. This instrument also enables heating or cooling temperature scans in the range between 15 °C and 65 °C. AT-cut quartz crystals with Au coating (diameter 14 mm, thickness 0.3 mm, quoted surface roughness < 3 nm, and resonant frequency 4.95 MHz) were used as solid surfaces. The Au-coated quartz sensors were cleaned for 5 min with a 5:1:1 mixture of Milli-Q water (resistance of 18.2 MΩ cm at 25 °C), ammonia, and hydrogen peroxide heated at 75 °C, then rinsed in Milli-Q water and dried with N_2_. Shortly prior to the beginning of the QCM-D measurements, the sensors were exposed to UV-light using a UV-ozone cleaner (Bioforce Nanosciences, Ames, Iowa, USA) for 15 min and subjected to contact angle measurements before their introduction into the QCM-D modules. The changes in Δ*f*/n and in Δ*D* were monitored at five different overtones (from third to eleventh). The running buffer and the lipid vesicles were injected into the QCM-D cells with a flow rate of 50 µL/min. The temperature stability was in the order of ± 0.02 °C around the set value. First, a baseline with pure HEPES buffer was established and afterward lipid vesicles were injected over the Au-coated sensor chips. After reaching a stable supported lipid membrane layer (vesicle or bilayer), temperature scans were carried out, upon heating and cooling, at a rate of 0.4 °C/min, maintaining a 60 min stabilization time between successive temperature ramps.

### 2.5. Atomic Force Microscopy (AFM)

AFM quantitative imaging (QI) and force spectroscopy measurements were performed using a JPK NanoWizard 4 BIO (Bruker, Nano GmbH, Berlin, Germany). All the measurements were done in HEPES buffer at room temperature (*T* ~ 20 °C). A MLCT-E tip (Bruker, Nano GmbH, Berlin, Germany) with quoted cantilever length of ~ 0.55 µm, resonance frequency ~ 38 kHz and nominal spring constant ~ 0.1 N/m was used. The AFM cantilever was calibrated in buffer against the clean glass slide composing the AFM liquid cell using the equipartition theorem [29]. After QCM-D measurements, samples were immediately transferred to the AFM liquid cell to minimize possible dewetting. QI images were taken on different image sizes using a pixel ratio of either 256 × 256 with the tip line rate of 1 Hz, cantilever speeds of 15 and 20 μm/s, and setpoint force of 0.2 nN. Force mapping was recorded by using force setpoints of 5 and 10 nN, with a speed of 1 µm/s.

### 2.6. Contact Angle Measurements

Water contact angle (WCA) measurements were performed using an Attension ThetaLite from Biolin Scientific (Gotheburg, Sweden) based on the sessile drop method. A small drop (3 μL) of Milli-Q water or diiodomethane was deposited onto clean, UV-ozone treated Au-coated sensors, and the shape of the drop formed on the surface was evaluated. The contact angle of the 3 μL droplet of ultrapure water was measured for 10 seconds using a recording speed of 20 frames/s. All contact angles were measured at room temperature. The UV-ozoned Au surfaces were hydrophilic (25° < WCA < 34°) as expected.

## 3. Results and Discussion

### QCM-D Results

**Figure 1:**
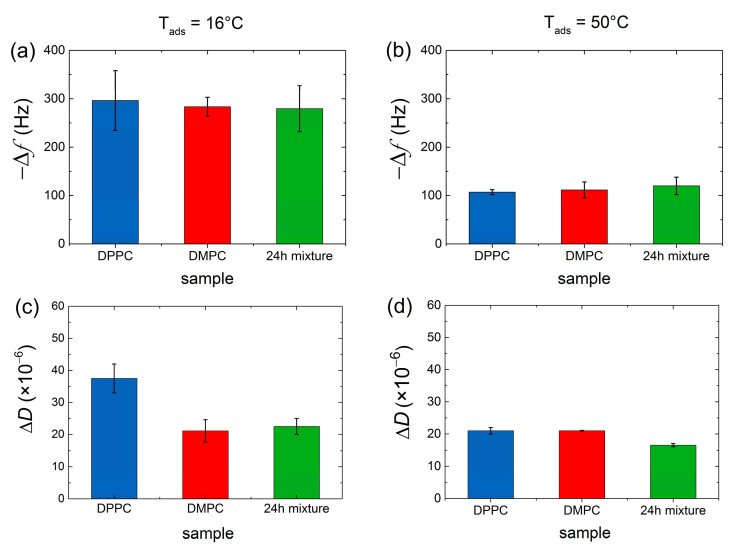
Frequency shifts Δ*f* (**a**,**b**) and dissipation shifts Δ*D* (**c**,**d**) for the third overtone observed during the adsorption of pure DMPC (red), pure DPPC (blue), and 24 h incubated DPPC/DMPC mixture (green) at *T*_ads_ = 16 °C (**a**,**c**) and *T*_ads_ = 50 °C (**b**,**d**) on Au-coated quartz sensors. Results obtained at *T*_ads_ = 32 °C can be found in SI (Figure S1).

DPPC and DMPC vesicle populations were mixed at equivalent mass concentration and incubated for different times (ranging between 0 to 48 h) and different temperatures (16 °C, 32 °C, and 50 °C) before adhesion onto Au-coated quartz surfaces heated at the same three temperatures. The fate of nanoscale lipid vesicles when adsorbing onto a solid sensor is dictated by the interplay between the adhesive energy from lipid–surface interactions and the opposing effect of bending the vesicle bilayer [30,31]. The former depends strongly on the nature of the surface and the latter on the phase of the vesicle constituent lipids. In general, when lipids are injected on Au surfaces, a monotonic frequency decrease (corresponding to mass increase) and a dissipation increase take place for reaching constant non-zero Δ*f* and Δ*D* plateau values, irrespective of the lipid phase, and indicating the formation of an intact vesicle layer [31]. Figure 1 indicates the plateau values of Δ*f* and Δ*D* responses (represented for the third overtone) after vesicle adsorption (pure lipids and mixed vesicle populations) on Au-coated quartz sensors at two incubation/adsorption temperatures (*T* = 16 and *T* = 50 °C). There was no indication of collective vesicle rupture, and continuous supported lipid bilayer was observed. However, as pointed out by Lind et al. [32], local vesicle rupture events and formation of bilayer patches cannot be ruled out. As a matter of fact, QCM-D is very sensitive to hydrodynamic (wet) mass, and the local, partial formation of SLBs is masked by the adsorption of vesicles on the top or in between the bilayer patches, as was later confirmed by AFM measurements.

At 16 °C, the bilayer envelopes of the vesicles are in the gel phase (*T* < *T_m_*), their bending modulus being *κ* ~ 10 × 10^−19^ J, which renders the vesicles quite stiff [33]. At *T* = 50 °C the bilayer envelope is in the liquid disordered phase, and the modulus attains a 10 times smaller value of *κ* ~ 1 × 10^−19^ J, making vesicles softer and more deformable upon adsorption, thus resulting in a smaller number of vesicles for similar surface coverage. Although vesicles do not rupture globally, attractive interactions to the Au surface induce deformation, and vesicles deform leading to a smaller thickness than in bulk, and even rupture locally, forming bilayer patches. At *T* = 32 °C, bilayer envelopes of DMPC vesicles are in their liquid phase, while the occurrence of periodic ripples in DPPC lipid bilayers is very likely. The frequency shifts plateau value Δ*f*_3_ ~ −179 Hz resulting from pure DMPC vesicles adsorption is in between those observed at *T* = 16 °C and *T* = 50 °C (see Figure S1). Pure DPPC and the mixed vesicle population display frequency shift values closer to the ones observed at *T* = 16 °C since DPPC lipids at *T* = 32 °C are mostly in the gel phase.

**Figure 2:**
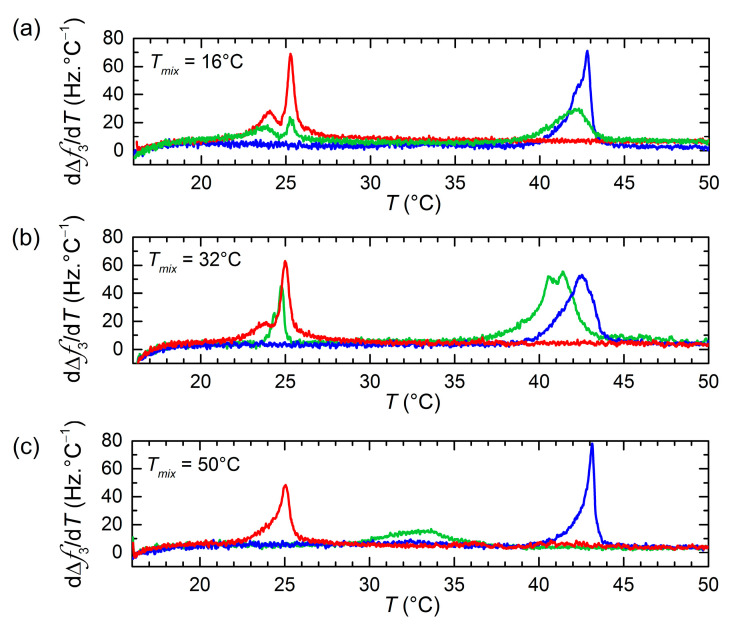
Temperature dependence of dΔ*f*_3_/d*T* (third overtone) upon the first heating for pure DMPC LUVs (red), pure DPPC LUVs (blue), and 24 h DPPC/DMPC mixture (green) adsorbed at *T* = 16 °C (**a**), *T* = 32 °C (**b**), and *T* = 50 °C (**c**) on Au-coated quartz surfaces.

After the vesicle/bilayer layer formation on the Au-coated sensors, subsequent heating and cooling cycles were carried out at a rate of 0.4 °C/min to assess the shifts in melting temperature resulting from lipid transfer between the two vesicle populations. Figure 2 displays the first-order temperature derivatives of Δ*f*(*T*) signals upon heating for vesicle populations of pure DPPC, pure DMPC, as well as DMPC and DPPC incubated for 24 h at different temperatures. The main phase transitions are identified by extrema (maxima) in the *d*Δ*f*(*T*)/*dT* responses. The main transitions for pure lipid DPPC or DMPC vesicles are characterized by large and rather sharp peaks located at temperatures in agreement with those reported in the literature using similar heating rates [31,34]. The main transitions were also observed upon cooling with a hysteresis of Δ*T_m_* ~ 2 to 3 °C, characteristic of first-order phase transitions [35] (an example of the hysteresis effect is shown in Figure S2 of the Supplementary Material). The phase transition of pure DMPC when incubated at low temperatures displays a double-peak behavior. The presence of the two peaks has been previously observed in the literature from calorimetric and QCM-D measurements, and it is a direct consequence of the extrusion process and related to structural transitions in the vesicle itself [36,37].

As regards the mixtures of DMPC and DPPC vesicle populations incubated for 24 h, a significantly different behavior is observed depending on the incubation temperature. At *T* = 16 °C, both DMPC and DPPC lipids are in the gel phase, and the location of the main transition peaks is practically unaffected. At *T* = 32 °C, DMPC is in the liquid-disordered phase, while DPPC is beginning the ‘gel to ripple’ phase transition, the majority of lipid molecules being in the gel phase. The location of the low-T peak remains unchanged, whereas the high-T peak is shifted toward lower temperatures, indicating DMPC lipid transfer into DMPC vesicles. At *T* = 50 °C, both lipids are in the liquid-disordered phase. After 24 h incubation, the low-T peak is not appearing anymore, whereas the high-T peak is significantly broadened and shifted toward lower temperatures.

**Figure 3:**
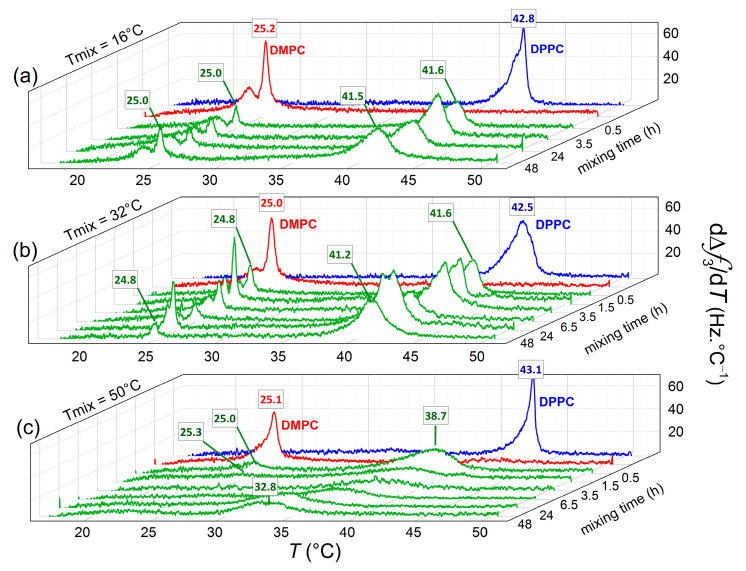
Dependence of the mixing time on the main phase transition parameters of adsorbed vesicles on Au-coated quartz surfaces. dΔ*f*/d*T* curves obtained upon heating demonstrate the phase transitions of samples at *T* = 16 °C (**a**), *T* = 32 °C (**b**), and *T* = 50 °C (**c**).

Figure 3 provides a three-dimensional overview of the phase transition peak evolution as a function of incubation time and temperature, with the aim to get more insights into the nature (symmetric or asymmetric) of lipid transfer between the vesicles adsorbed onto the Au surfaces. At the beginning of the incubation period (*t* ~ 0.5 h), two clear peaks can be distinguished for vesicle populations incubated at all three temperatures. At *T* = 16 °C (panel a) and *T* = 32 °C (panel b), the location of the transition temperatures of the mixed vesicle populations remains close to their pure lipid counterparts, while the peak intensities are reduced. The reduction in peak intensity can be attributed to (i) limited lipid transfer taking place and transitions becoming broader and (ii) local vesicle rupture occurring upon adsorption (the peak intensity scales with the mass adsorbed, including coupled water trapped within intact vesicles). At *T* = 50 °C (panel c), the low temperature peak is located very close to the phase transition of pure DMPC, while the high temperature peak is significantly shifted as compared with pure DPPC melting temperature (Δ*T_m_* ~ −4.4 °C). With increasing incubation time, the original transition of the DPPC vesicle population is shifted to lower temperatures, whereas the low temperature peak remains at the same location and vanishes with time (see Figure S3 in the Supplementary Materials). The DMPC vesicle population is continuously depleted of its lipids until the vesicles become unstable and equimolarity is reached. This process was completed from 24 h incubation after which the no significant shifts in the transition peak were observed. As a matter of fact, the temperature interval where the transition takes place shows a good agreement with previously reported transitions of equimolar mixtures of DMPC and DPPC vesicles formed with lipids previously mixed [38].

**Figure 4:**
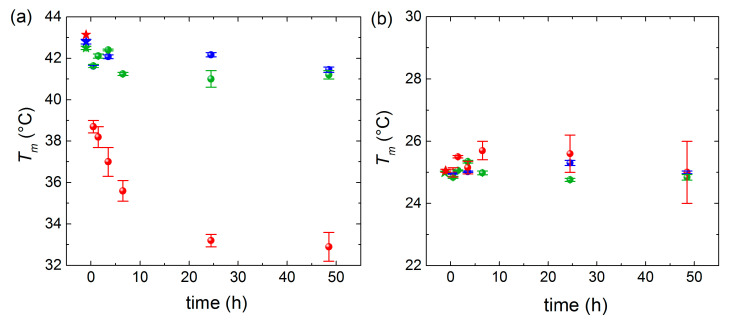
Dependence of the incubation time on *T_m_* values extracted from (**a**) high-*T* peaks and (**b)** low-*T* peaks (blue points for *T* = 16 °C, green points for 32 °C, red points for 50 °C). Stars represent *T_m_* of pure DPPC vesicle (**a**) and pure DMPC vesicles (**b**), which are used as reference.

For the sake of clarity regarding the melting temperature shifts observed in Figure 3, we plotted the mixing time dependence of the main phase transitions at the three incubation temperatures for the high-*T* (Figure 4a) and low-*T* peaks (Figure 4b). At *T* = 16 °C and *T* = 32 °C, the displayed time-dependent *T_m_* values deviate only slightly from the ones of pure lipids. Conversely, at *T* = 50 °C, a continuous decrease is observed for the high-T peak, while the low-T peak displays a very mild increase, indicating that asymmetric lipid transfer takes place from DMPC vesicles (donor) toward DPPC vesicles (acceptor).

**Figure 5:**
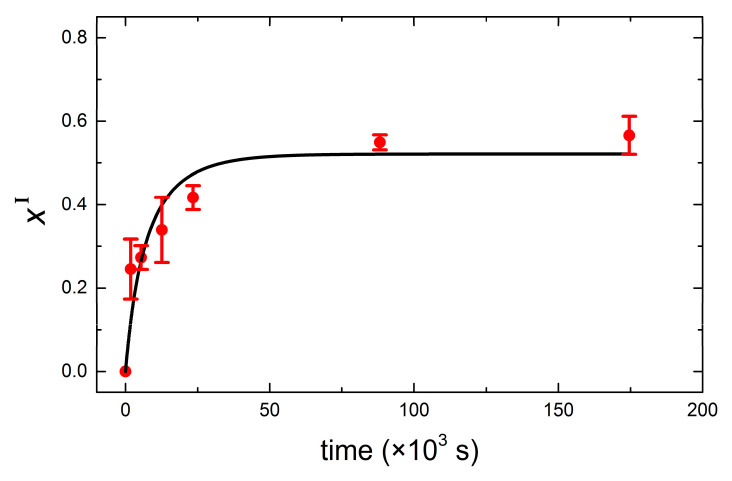
Mole fraction of DMPC in vesicle population I (DPPC vesicles) as a function of time. Red solid dots are experimental points, and the black solid line is the fit to Equation (3).

**Figure 6:**
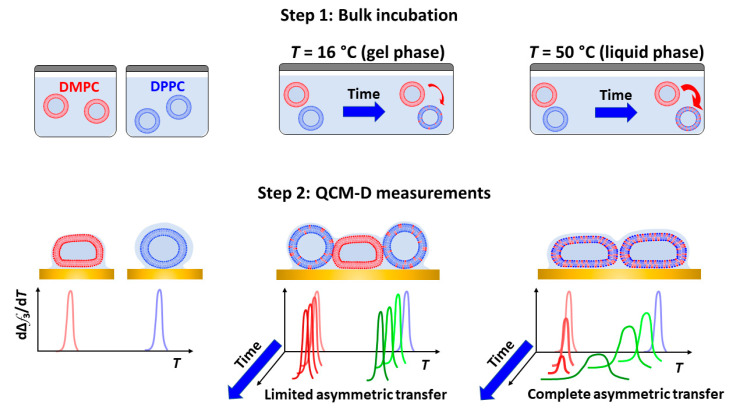
Schematic representation of lipid transfer detected by QCM-D nanoviscosity measurements.

In order to get insights into the kinetics of lipid transfer, we analyzed our results in the framework of a kinetic model introduced by Thilo [39] and later applied by Bayerl et al. [40] on DMPC–DPPC exchange of small vesicles using calorimetric measurements. Bayerl et al. reported that in the case of small sonicated vesicles (diameter ~ 60 nm), lipid transfer took place by monomer transfer from DMPC to DPPC vesicles, whereas larger vesicles (200 nm < diameter < 900 nm) prepared by detergent dialysis exchanged lipids mainly by vesicle fusion. The conclusions were based on the shape and evolution of transition peaks from heat capacity curves of vesicle populations incubated at *T* = 45 °C. The former displayed *T_m_* shifts of the high-T peak, while the latter both high-*T* and low-*T* remained non-shifted with time, with a broader peak located in between the unshifted peaks arising and coexisting at longer incubation times. Our results coincide with the results previously reported for small sonicated vesicles; thus, we conclude that lipid transfer in our systems takes place rather by monomer transfer from DMPC to DPPC vesicles. During spontaneous lipid transport, DMPC first desorbs from the donor vesicle, diffuses through the solvent (buffer), and enters the DPPC acceptor membrane. The model of Thilo assumes that (a) the rate-limiting step is the monomer desorption from the donor vesicles, and (b) the rate at which lipid monomers in the bulk solution are taken by the acceptor vesicles is proportional to the product of the monomer concentration and the total bilayer surface area in a unit volume. It considers two vesicle populations I and II and assumes that at time zero (*t* = 0) the former contains only DPPC and the latter DMPC. After an incubation time *t*, the transition temperatures *T^I^*(*t*) and *T^II^*(*t*) of vesicle populations I and II can be expressed as:

(1) $$T^{I}(t)=x^{I}(T_{m}^{DMPC}-T_{m}^{DPPC})+T_{m}^{DPPC}$$

(2) $$T^{II}(t)=x^{II}(T_{m}^{DMPC}-T_{m}^{DPPC})+T_{m}^{DPPC}$$

where *x^I^* and *x^II^* are the molar fractions of DMPC in vesicle populations I and II, respectively. From the values of *T^I^*(*t*) and *T^II^*(*t*), the off rate constants of DMPC and DPPC dissociated from their original vesicle populations can be obtained:

(3) $$x^{I}(t)=\frac{1-e^{-yt}}{\frac{1}{r}-(1-k_{DPPC}^{off}/k_{DMPC}^{off})e^{-yt}}$$

(4) $$x^{II}(t)=\frac{(1-r)k_{DMPC}^{off}e^{-yt}+rk_{DPPC}^{off}}{(1-r)(k_{DMPC}^{off}-k_{DPPC}^{off})e^{-yt}+k_{DPPC}^{off}}$$

with $y=rk_{DPPC}^{off}+(1-r)k_{DMPC}^{off}$ and r=[DMPC]/[(DMPC]+[DPPC]). In our particular case, *r* = 0.52 and $k_{DPPC}^{off}=0$, assuming asymmetric unidirectional lipid transfer. Our assumption is based on the fact that the lipid desorption from the donor vesicles is the rate-limiting step and determines the activation energy of lipid transfer. As recently reported by Rogers et al. [41], the membrane hydrophobicity dictates the activation energy for lipid transfer. The energy cost for desorption from the donor vesicle increases as the lipid acyl chain increases and membrane order increases. Experimentally, activation energies for interbilayer monomer transfer and transbilayer (flip-flop) transfer can be accurately determined by time-resolved small angle neutron scattering, as used by Nakano et al. for symmetric transfer between DMPC LUVs [42]. In our particular case, DPPC being longer than DMPC and in the gel phase, its activation energy is larger and thus less likely for monomer desorption. Figure 5 displays the calculated mole fraction of DMPC into DPPC vesicles incubated at 50 °C as a function of time, together with its corresponding fit to Equation (3). The resulting off rate constant $k_{DMPC}^{off}=(16\pm 4)\;\times \;10^{-5\;}s^{-1}$ and half-time for 50% lipid transfer $t_{1/2}=1.46\pm 0.04\;h$ agree very well with reported calorimetric results by Bayerl et al. As expected, the off rates obtained for samples incubated at 16 °C and 32 °C are considerably reduced, and accordingly, the half-times are dramatically increased. At *T* = 16 °C, both DMPC and DPPC lipids are in the gel phase, and transfer is limited by the decreased desorption rate of DMPC monomers from the donor vesicles. At *T* = 32 °C, DMPC is in the liquid disordered phase, and thus a faster DMPC monomer desorption from the donor vesicles occurs. However, the transfer is very likely limited by the reduced monomer insertion rate due to the fact that DPPC is mostly in the gel phase. For the sake of clarity, a summary of the (asymmetric) transfer detected by QCM-D is schematically depicted in Figure 6. The results at low temperatures agree qualitatively with calorimetric results in that transfer is significantly reduced when lipids are in the gel phase. A quantitative comparison is out of the scope of this paper since additional data at higher incubation times (*t* > 48 h) would be necessary in order to achieve precise fits for mixing at 32 °C and 16 °C.

**Figure 7:**
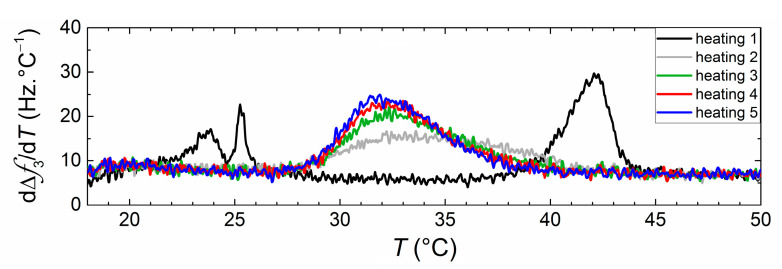
dΔ*f*_3_/d*T* curves obtained upon successive heatings of the DPPC and DPPC vesicle dispersions incubated for 24 h at *T* = 16 °C.

After the first thermal (heating and cooling) cycle, subsequent cycles were carried out to assess the effect of crossing the phase transition on the net transfer. Figure 7 shows the dependence of phase transition behavior upon thermal cycling for vesicle dispersions of DMPC and DPPC incubated at 16 °C during 24 h. During the first heating, two peaks corresponding to the melting of two independent vesicle populations could be clearly observed, indicating the absence of lipid transfer at low temperatures. During the second heating a broad single peak appears, which becomes sharper with subsequent thermal cycles, indicating that crossing the main transition accelerates the lipid transfer between the two vesicle populations. A similar pattern of behavior can be seen for vesicles incubated at 32 °C (see Figure S4 in the Supplementary Materials). Since during the thermal cycles, vesicles are adsorbed on the Au surface, we are inclined to think that transfer occurs in this case by vesicle fusion, induced by the change in mechanical properties of adsorbed vesicles (changing from a stiffer to a softer state). Vesicle populations incubated at 50 °C display no significant changes upon thermal cycling since transfer until equimolarity had already occurred in bulk (see Figure S4 in the Supplementary Materials).

Immediately after the thermal cycles were carried out in the QCM-D flow cells, the Au-coated QCM-D sensors with the supported lipid layers were transferred to an AFM liquid cell. The QCM-D sensors were kept submerged in buffer at all times to ensure that the lipid layers were always hydrated. The surface coverage of the Au-sensors with the lipid layers was assessed by QI-mode imaging at low force setpoint, followed by force mapping at higher force setpoints. A freshly cleaned gold-coated QCM-D sensor was used as reference. Figure S5 of the Supplementary Materials displays the topography of a bare Au sensor (a) as well as pure DMPC, pure DPPC, and adsorbed vesicles incubated at 50 °C for 24 h in HEPES buffer (b). The bare Au surface is rather flat (RMS roughness = 0.95 ± 0.05) with polycrystalline texture with grains of lateral size ~ 80 nm as recently shown [43]. Surfaces with adsorbed lipid layers appear more inhomogeneous, displaying both supported lipid bilayers and intact adsorbed vesicle layers. As reported by Lipowsky and coworkers [44], homogeneous coverage of lipid layers is limited on polycrystalline surfaces, while it can take place on large Au grains with atomically flat (111) terraces. Unlike layers formed onto atomically flat surfaces such as mica, the underlying substrate roughness prevents distinguishing whether the formed layers are bilayers, monolayers, or multilayers by solely inspecting the height measured of the layers. The presence of each type of layer was evaluated by the shape of force curves recorded at different spots of each sample. Samples containing pure DMPC consisted of 64% bilayers, 26% intact vesicles, and 10% multilayers. Intact vesicles could have fused and ruptured during the thermal cycling by thermal stress. Pure DPPC consisted of 12% bilayers, 85% intact vesicles, and 4% multilayers. Vesicle populations of DMPC and DPPC incubated 24 h at 50 °C displayed a composition of 45% bilayers, 50% intact vesicles, and 5% multilayers. For simplicity, we will only evaluate the nanomechanical properties of supported lipid bilayers.

**Figure 8:**
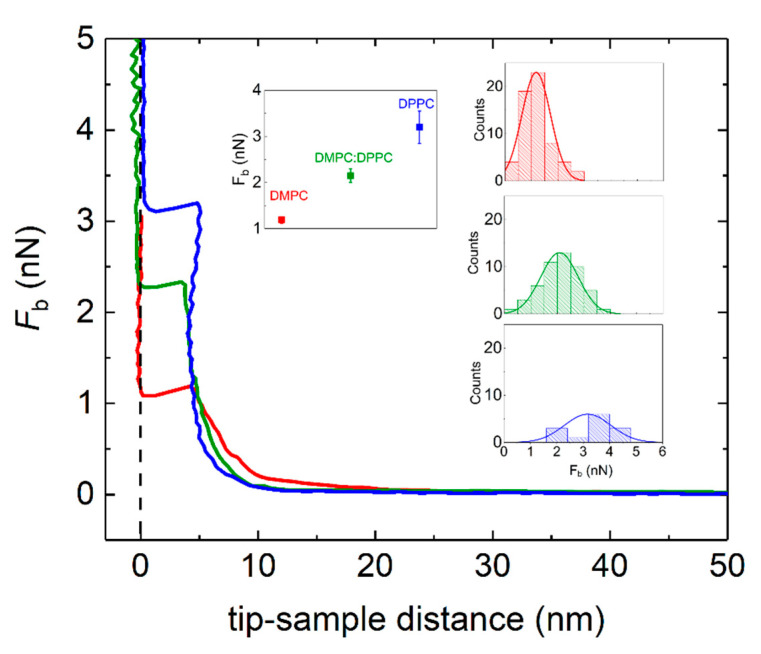
Approach force-distance curves on DMPC (red solid line), DMPC:DPPC (green solid line), and DPPC (blue solid line) supported lipid bilayers on Au-coated QCM-D sensors. Inset: Corresponding histograms taken from a given number of force-distance curves.

Figure 8 displays representative force curves as a function of the tip-sample separation distance *d* during approach. The zero-separation distance *d* = 0 is defined as the point where the tip comes into hard contact with the surface. Upon approaching the AFM tip to the lipid-covered Au surface, no interaction is observed until *d* ~ 15 nm tip-surface vertical separation distance, when repulsive interactions between the tip and the lipid covered surface take over. From that point, the supported lipid layer is elastically compressed until the tip punches through the layer and enters into contact with the Au surface. The discontinuity in the approaching force distance curve stands as a token of the penetration of the AFM tip through the lipid bilayer. The vertical force at which this discontinuity takes place corresponds to the maximum force that the bilayer is able to withstand before breaking, commonly referred to as the breakthrough force (*F*_b_) [45,46]. The tip-sample distance at which penetration occurs relates to the thickness of the lipid layer formed. In all cases, the average tip-sample distance falls within 4 to 5 nm, which agrees well with the typical thickness of compressed supported lipid bilayers. Interestingly, the average value breakthrough force *F*_b_ shows a clear trend, the system where lipid transfer has taken place displaying an intermediate value between pure DMPC and pure DPPC (see inset Figure 7). The corresponding statistical analysis for the jump thickness can be found in Figure S6 of the Supplementary Material. It is worth mentioning that all samples were measured at room temperature (*T* ~ 20 °C), where both DMPC and DPPC lipid bilayers are in the gel phase. The larger value of *F*_b_ for DPPC can be ascribed to the increased hydrophobic interactions due to longer alkyl chains.

## 4. Conclusions

In this work we have demonstrated the capability of QCM-D to monitor the dynamics of lipid transfer between nanoscale-sized lipid vesicles consisting of zwitterionic lipids differing only in two ethyl groups—namely, DMPC and DPPC. Lipid transfer kinetics was assessed by time-dependent changes in viscoelastic properties of the supported membrane layers during their main phase transition. Lipid transfer proceeds in an asymmetric manner, from donor (DMPC) to acceptor (DPPC) vesicles by lipid monomer transfer. Our measurements reflect the influence of lipid physicochemical and related mechanical properties on the kinetics of lipid transfer. When both types of vesicles are in the gel phase, transfer is very slow and limited by the reduced desorption and insertion rates. When both types of vesicles are in the liquid disordered phase, transfer proceeds faster and kinetic rates agree well with those obtained by calorimetry. Thermal cycling through the transition accelerates the transfer, even for vesicle populations that were originally incubated at gel phases. Complementary AFM measurements provide nanomechanical signatures of supported lipid bilayers where transfer has taken place. Homogeneously mixed layers display breakthrough forces in between those observed for their pure short-chain and long-chain counterparts.

Overall, lipid transfer kinetics assessed by changes in viscoelastic properties of the adsorbed lipid layers are in agreement with calorimetry measurements and demonstrate the potential nano of QCM-D for detecting and quantifying lipid transfer between zwitterionic nanoscale-sized vesicles with high sensitivity and reduced amounts of biomolecules. We anticipate additional measurements of transfer in situ to shed more light on the role of layer topography in transfer kinetics.

## Figures and Tables

**Table 1:** Hydrodynamic mean diameters and polydispersity indexes (PI) obtained by DLS for the DPPC and DMPC vesicle dispersions used in this work. The number of performed measurements per sample is *n* = 4. The calculated errors are the standard deviation of the average values based on several independent solutions.

Lipid	Temperature (°C)	Mean Diameter (nm)	PI
DPPC	16	119 ± 6	0.12 ± 0.06
DMPC	16	118 ± 9	0.05 ± 0.01
DPPC	32	110 ± 3	0.08 ±0.01
DMPC	32	120 ± 3	0.08 ± 0.01
DPPC	50	130 ± 5	0.07 ± 0.02
DMPC	50	126 ± 7	0.09 ± 0.01

## Data Availability

The data presented in this study are available from the corresponding author upon request.

## Supplementary Materials

The following are available online at https://www.mdpi.com/article/10.3390/nano11051087/s1. Figure S1: Frequency shifts (a) and dissipation shifts (b) observed during the adsorption of pure DMPC (red), pure DPPC (blue), and 24 h DPPC/DMPC mixture (green) at 32 °C on Au quartz sensors; Figure S2: Temperature dependence of dΔ*f*_3_/d*T* (3rd overtone) upon cooling and heating for pure DMPC LUVs and pure DPPC LUVs adsorbed at 50 °C on Au-coated quartz surfaces; Figure S3: Dependence of the mixing time (30 min, 1 h 30 min, 3 h 30 min, 6 h 30 min, 24 h, or 48 h) on the main phase transition temperature of adsorbed vesicles on Au-coated quartz surfaces. dΔ*f*/d*T* curves obtained upon heating demonstrate the phase transitions of samples incubated at 50 °C (c). Figure S4: dΔ*f*/d*T* curves obtained from successive heatings of the DPPC and DMPC vesicle dispersions incubated for 24 h at *T*_inc_ = 16 °C, 32 °C, and 50 °C; Figure S5: Height measured of a bare Au sensor (a) as well as pure DMPC, pure DPPC, and adsorbed vesicles incubated at 50 °C for 24 h in HEPES buffer (b); Figure S6: Statistical analysis of the jump thickness upon lipid bilayer perforation. The histograms correspond to a given number of force curves taken on DMPC (red solid line), DMPC:DPPC (green solid line), and DPPC (blue solid line) supported lipid bilayers on Au-coated QCM-D sensors.
