# Supplementary material for: Asymmetric Lipid Transfer between Zwitterionic Vesicles by Nanoviscosity Measurements
Source: Nanomaterials (Basel). 2021 Apr 22;11(5):1087. doi: 10.3390/nano11051087 (PMC8145678; doi:10.3390/nano11051087)
Supplement: Supplementary file 1 [file nanomaterials-11-01087-s001.zip › nanomaterials-1188197-supplementary.pdf]

Asymmetric lipid transfer between zwitterionic vesicles by nanoviscosity measurements

Laure Bar [1], George Cordoyiannis [2], Shova Neupane [3], Jonathan Goole [4], Patrick Grosfils [5], and Patricia Losada-Pérez [1,*]

[1] Experimental Soft Matter and Thermal Physics group (EST), Department of Physics, Université Libre de Bruxelles, Boulevard du Triomphe CP223, 1050 Brussels, Belgium

[2] Condensed Matter Physics Department, Jožef Stefan Institute, Jamova 39, 1000 Ljubljana, Slovenia

[3] Physical Chemistry of Surfaces Group, Institut de Recherche de Chimie Paris (IRCP), 11 rue Pierre et Marie Curie, 75005 Paris, France

[4] Laboratory of Pharmaceutics and Biopharmaceutics, Université libre de Bruxelles, Campus de la Plaine, CP 207, Boulevard du Triomphe, Brussels, 1050, Belgium

[5] Center for Nonlinear Phenomena and Complex Systems, Department of Physics, Université Libre de Bruxelles, Boulevard du Triomphe CP223, 1050 Brussels, Belgium

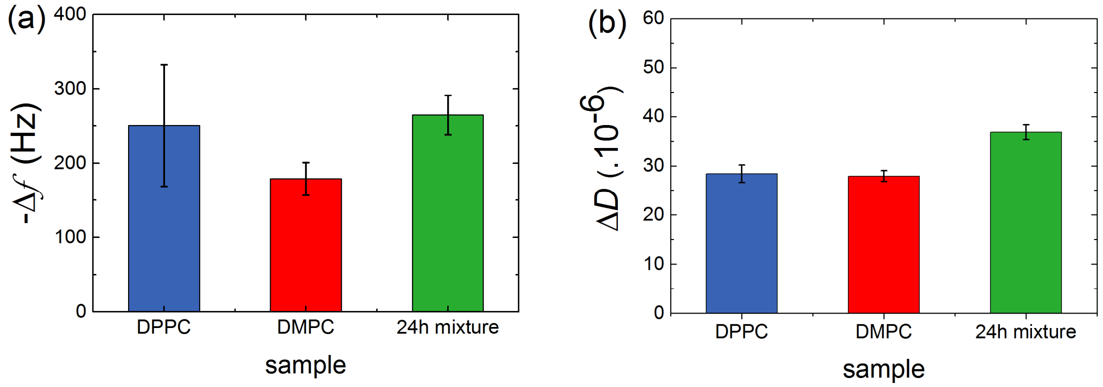

Figure S1. Frequency shifts (a) and dissipation shifts (b) for the third overtone observed during the adsorption of pure DMPC (red), pure DPPC (blue) and 24h DPPC/DMPC mixture (green) at 32°C on Au quartz sensors.

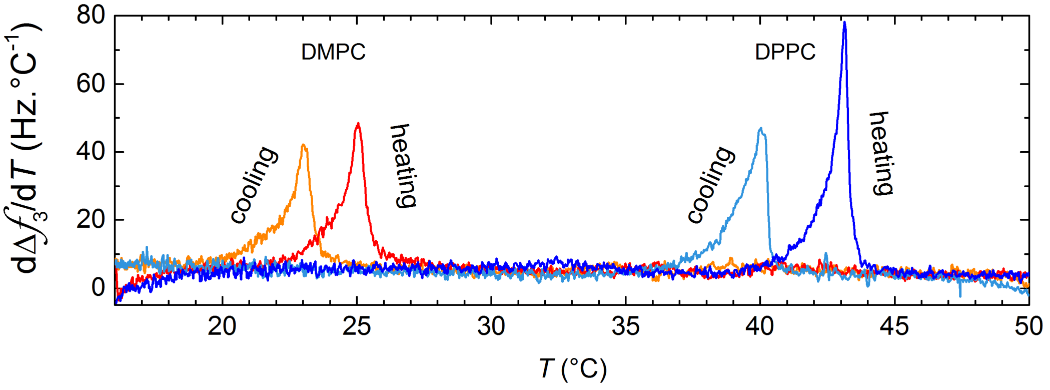

Figure S2. Temperature dependence of dΔf/dT (3rd overtone) upon cooling and heating for pure DMPC LUVs and pure DPPC LUVs adsorbed at 50°C on Au-coated quartz surfaces. Upon cooling the phase transitions occur at lower temperatures than upon heating. Here, $|\Delta T_m$ (DPPC)$|$ = 3.1° C, and $|\Delta T_m$ (DMPC)$|$ = 2.1° C.

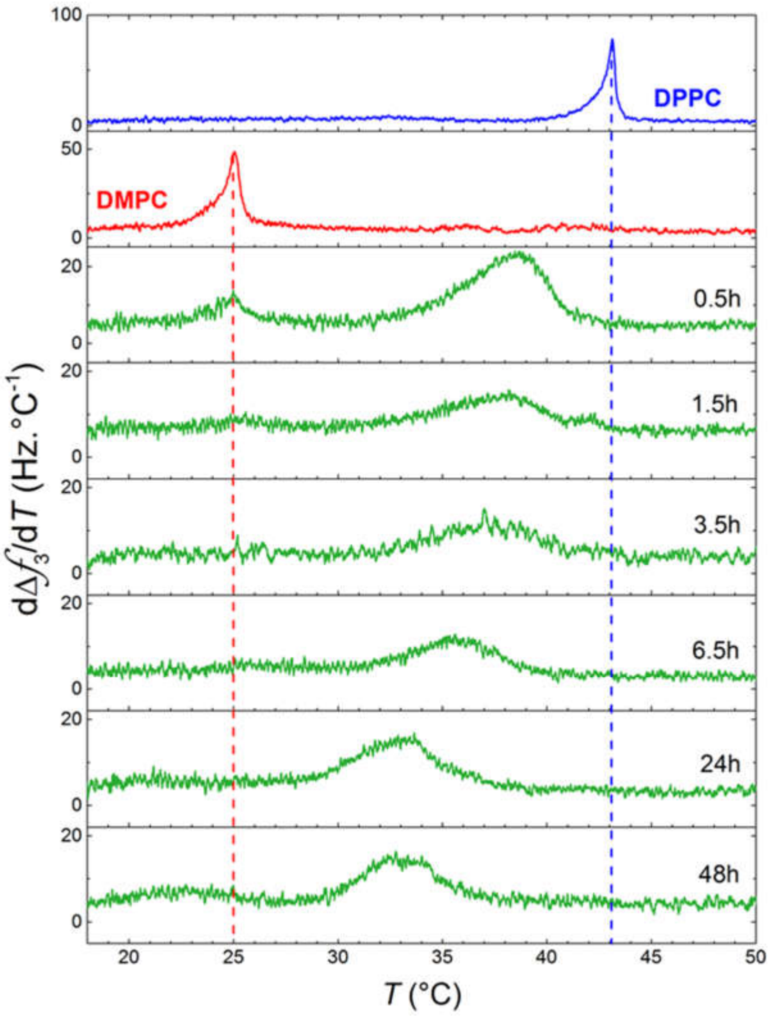

Figure S3. Dependence of the mixing time (30 min, 1h30, 3h30, 6h30, 24h, or 48h) on the main phase transition temperature of adsorbed vesicles on Au-coated quartz surfaces. dΔf_3/dT curves (for the 3rd overtone) obtained upon heating demonstrate the phase transitions of samples incubated at 50 °C.

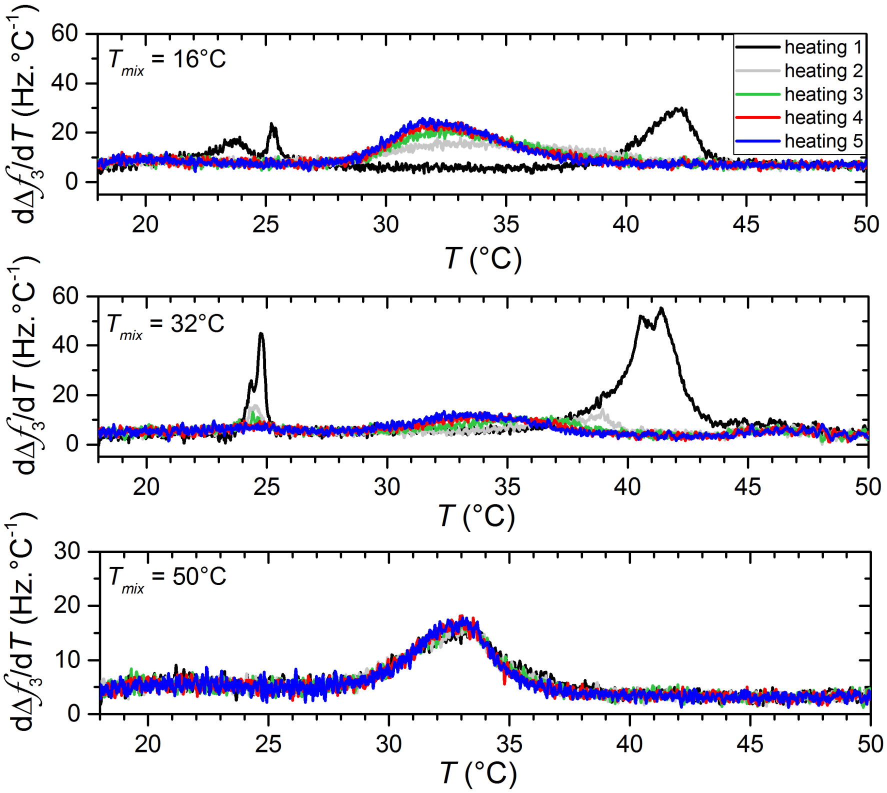

Figure S4. dΔ*f*/d*T* curves obtained from successive heatings of the DPPC and DMPC vesicle dispersions incubated for 24h at *T* = 16 °C (top layer), *T* =32 °C (middle layer), and *T* = 50 °C (bottom later).

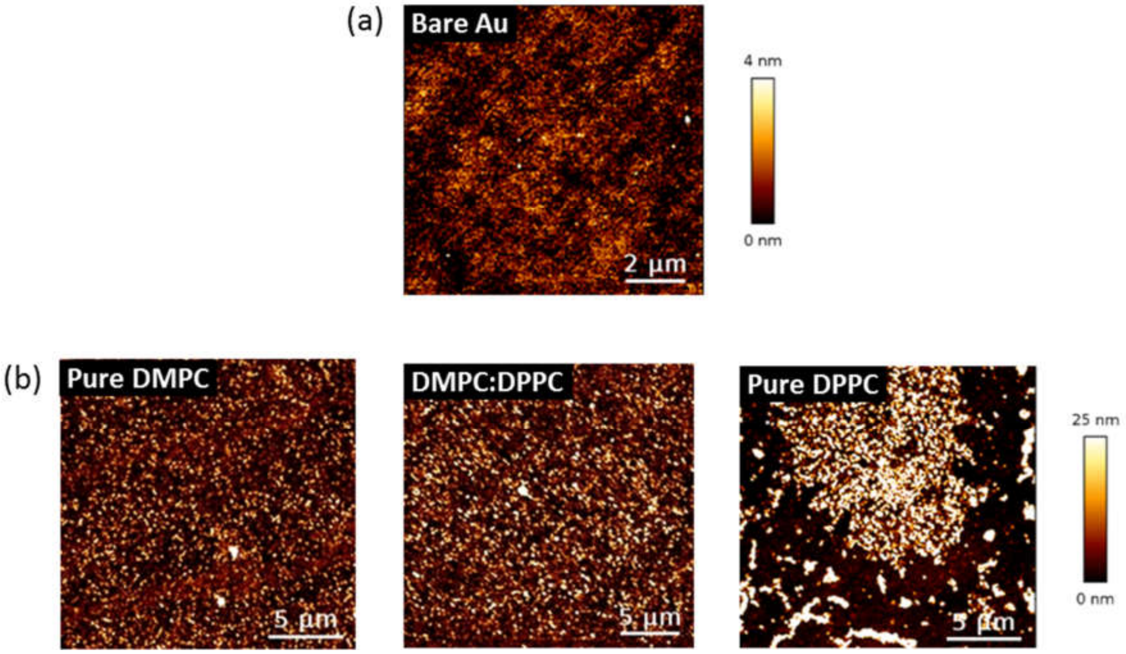

Figure S5. AFM height measured images taken on QCM-D quartz gold sensors : (a) is a bare Au used as reference, (b) is from left to the right pure DMPC, DMPC:DPPC incubated at 50 °C for 24 h and pure DPPC coated surfaces after thermal cycles.

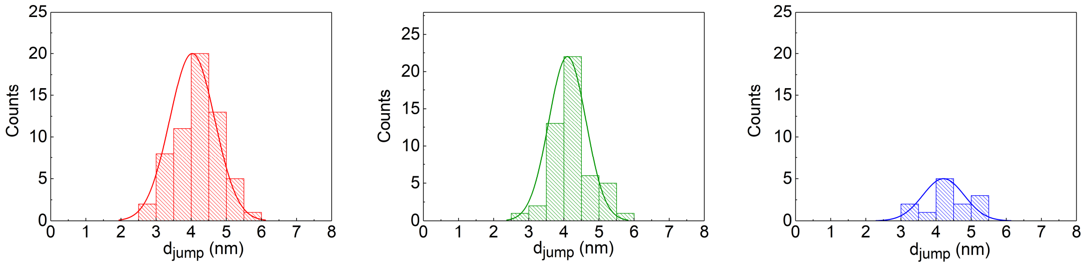

Figure S6. Statistical analysis of the jump thickness upon lipid bilayer perforation. The histograms correspond to a given number of force curves taken on DMPC (red solid line), DMPC:DPPC (green solid line) and DPPC (blue solid line) supported lipid bilayers on Au-coated QCM-D sensors.
